# Effects of Endocrine Therapy on Cognitive Function in Patients with Breast Cancer: A Comprehensive Review

**DOI:** 10.3390/cancers14040920

**Published:** 2022-02-12

**Authors:** Lucy R. Haggstrom, Janette L. Vardy, Emma-Kate Carson, Davendra Segara, Elgene Lim, Belinda E. Kiely

**Affiliations:** 1 Campbelltown Hospital, Therry Road, Campbelltown, NSW 2560, Australia; lucy.haggstrom@health.nsw.gov.au (L.R.H.); emmakate.carson@health.nsw.gov.au (E.-K.C.); 2 Faculty of Medicine and Health, University of Sydney, Camperdown, NSW 2006, Australia; janette.vardy@sydney.edu.au; 3 Concord Cancer Centre, Concord Repatriation and General Hospital, Concord, NSW 2139, Australia; 4 Concord Clinical School, University of Sydney, Concord, NSW 2139, Australia; 5 St Vincent’s Clinical School, University of New South Wales, Darlinghurst, NSW 2010, Australia; david.segara@svha.org.au; 6 Garvan Institute of Medical Research, Darlinghurst, NSW 2010, Australia; 7 NHMRC Clinical Trials Centre, University of Sydney, Camperdown, NSW 2050, Australia

**Keywords:** breast cancer, endocrine therapy, cognitive dysfunction

## Abstract

**Simple Summary:**

Many persons diagnosed with breast cancer are treated with endocrine therapy and will experience the side effects of endocrine therapy. Cognitive adverse effects of endocrine therapy are increasingly being recognised, and can significantly affect quality of life, adherence and treatment outcome. This review aims to discuss the nature of cognitive dysfunction associated with endocrine therapy, the mechanisms underpinning its development, and evidence-based management strategies.

**Abstract:**

Endocrine therapy forms the backbone of systemic therapy for the majority of persons with early and late-stage breast cancer. However, the side effects can negatively affect quality of life, and impact treatment adherence and overall oncological outcomes. Adverse effects on cognition are common, underreported and challenging to manage. We aim to describe the nature, incidence, risk factors and underlying mechanisms of endocrine therapy-induced cognitive dysfunction. We conducted a comprehensive literature review of the studies reporting on cognitive dysfunction associated with endocrine therapies for breast cancer. We also summarise prevention and treatment strategies, and ongoing research. Given that patients are taking endocrine therapies for longer durations than ever before, it is essential that these side effects are managed pro-actively within a multi-disciplinary team in order to promote adherence to endocrine therapy and improve patients’ quality of life.

## 1. Introduction

Worldwide, breast cancer is the most common cancer diagnosed in women, with a lifetime incidence of 5–20%, and it is the leading cause of cancer-related death [1,2]. Approximately 70–75% of all invasive breast carcinomas are oestrogen receptor positive (ER+) [3]. Endocrine therapy forms the backbone of treatment of ER+ breast cancer, and includes selective oestrogen receptor modulators (e.g., tamoxifen), selective oestrogen receptor degraders (e.g., fulvestrant), aromatase inhibitors (e.g., letrozole, anastrazole, exemestane) and ovarian function suppression (e.g., goserelin). While each of these treatments has a different mechanism of action, menopausal symptoms and cognitive side effects have been reported with most of these agents.

The treatment of hormone receptor positive breast cancer has evolved significantly in recent years. Many advances have contributed to declines in mortality attributable to breast cancer, and survival is predicted to increase further in the coming years [4]. In both early and advanced ER+ breast cancer, patients are remaining on endocrine-based therapies for longer periods of time. In early-stage breast cancer, the recommended duration of adjuvant endocrine therapy now extends for up to 10 years, in order to reduce the rate of late relapse and death from breast cancer [5,6]. In addition, ovarian function suppression may be used in pre-menopausal women with high-risk breast cancer to further lower oestrogen levels, and reduce the risk of recurrence and death [7,8]. In advanced-stage breast cancer, combining endocrine therapy with molecular therapies targeting intracellular pathways, such as cyclin-dependent kinase (CDK) 4/6 inhibitors, phosphatidylinositol 3-kinase (PI3K) inhibitors and mammalian target of rapamycin (mTOR) inhibitors, have been highly efficacious when used in combination with endocrine therapy as a backbone [9,10]. For example, CDK 4/6 inhibitors combined with endocrine therapy are now standard first line treatment for metastatic hormone receptor positive, HER2 negative breast cancer based on significant improvements in progression free survival and overall survival [11,12,13].

Given people with ER+ breast cancers are continuing endocrine therapies for longer durations, it is important to recognise the toxicities of endocrine therapy and the impact these toxicities can have on quality of life and daily functioning. Cognitive side effects from endocrine therapy are common but often not reported by patients, or recognised and treated by clinicians. For example, in a survey of 2296 women within situ or invasive breast cancer, only 37% of women experiencing cognitive symptoms discussed their symptoms with a health professional, and of these women, only 30% received treatments for their symptoms, including 8% of which were alternative treatments [14]. These side effects can have substantial impacts on patients’ abilities to carry out normal activities of daily living, adversely impact their quality of life, and increase one’s likelihood to discontinue therapy, which may increase the risk of recurrent disease or death [15,16]. Effective strategies to minimise endocrine therapy side effects and improve treatment adherence are therefore needed. While cancer-related cognitive impairment is becoming more recognised as a phenomenon, most literature has focused on the effects of chemotherapy; the impact of endocrine therapy has been less studied. The aim of this review is to describe the nature of cognitive dysfunction associated with endocrine therapy, as well as the underlying mechanisms, management strategies and areas for future research.

## 2. Materials and Methods

We searched the Pubmed database (accessed on 6 November 2021) for studies published until that date. The search strategy was as follows: “breast cancer” or “breast neoplasm” or “breast tumour*” AND (“endocrine therapy” OR “fulvestrant” OR “tamoxifen” OR “aromatase inhibitor*” OR anastrozole OR letrozole OR exemestane) AND “cogniti*. Observational, interventional and animal studies were included. Studies exploring the frequency of cognitive dysfunction where all patients had received chemotherapy before endocrine therapy were excluded owing to confounding effects of chemotherapy on cognition, however management and intervention studies were included, given the limited data available regarding this. Studies that compared participants receiving endocrine therapy alone with those receiving chemotherapy alone or combined with endocrine therapy were included. Studies evaluating endocrine therapy in combination with therapies other than chemotherapy were included to present preliminary information, given the unknown effects of these agents on cognition. The review was limited to articles in English, with full text data published. Articles were screened based on title and abstract, and the remaining full text articles were screened for relevance. Reference lists from studies and reviews were also hand-searched. The search revealed 557 articles, of which 72 were relevant for inclusion in the study.

## 3. Results

### 3.1. Impact of Oestradiol on Cognition

Pre-clinical research has highlighted the important influence oestrogen has on cognition. This occurs mostly via 17β-oestradiol, the most potent and predominant form of oestrogen in women, which can be synthesised de novo from cholesterol in the brain [17]. Most 17β-oestradiol signalling occurs through two receptors, oestrogen receptor alpha (ERα) and beta (ERβ), which, in addition to being expressed in the breast and hormone receptor positive breast cancer, are distributed throughout regions important in learning, memory and executive function, such as the hypothalamus, amygdala, hippocampus and dorsolateral prefrontal cortex [18,19,20]. Aromatase, an enzyme that catalyses the conversion of androgens to oestradiol, is widely distributed throughout the cerebral cortex, hippocampus, hypothalamus and midbrain [21,22]. Changes in aromatase activity due to pharmacological inhibition can therefore influence local oestradiol synthesis and significantly influence cognitive function [22]. For instance, in rat models, pharmacological inhibition of aromatase has been demonstrated to reduce dendritic spine density on synapses in the hippocampus and influence axonal growth [21,23]. Similarly, in rat models, spatial working memory has been demonstrated to be impaired by oophorectomy [24], and improved by oestradiol replacement [25,26]. Oestradiol also modulates multiple other neurotransmitters, such as noradrenaline, dopamine, 5-hydroxytryptamine, acetylcholine and gamma-aminobutyric acid, which can influence cognition [25,27].

Certain cognitive processes are particularly sensitive to the effects of oestradiol, likely due to the distribution of oestrogen receptors and aromatase within the brain. These processes include verbal memory, which is influenced by hippocampal function [28], and executive functions, such as response inhibition, working memory, problem solving, reasoning and behavioural monitoring, which are mediated primarily through the prefrontal cortex [29].

These findings in animal models have been supported by human studies using functional neuroimaging. For instance, using fluorodeoxyglucose positron emission tomography (FDG PET), Eberling et al. (2004) studied cerebral function in postmenopausal women receiving tamoxifen (*n* = 10), healthy postmenopausal women without breast cancer receiving hormone replacement therapy (*n* = 15) and healthy controls (*n* = 15). They observed that tamoxifen use was associated with decreased glucose metabolism in the dorsolateral and inferior frontal lobes, while hormone replacement therapy was associated with the highest metabolism in the three groups [30]. In another case-control study, 35 women with breast cancer receiving an aromatase inhibitor and 35 healthy controls underwent detailed neuropsychological testing and FDG PET at baseline prior to commencing aromatase inhibition and 6 months after commencement. While no significant changes in cognition were observed, there were significant changes in metabolic activity in the patients receiving aromatase inhibitors, particularly in the medial temporal lobes [31]. This finding is in keeping with the animal models that have demonstrated the high concentration of aromatase in the hippocampus [21]. Furthermore, in a randomised controlled trial in which perimenopausal and postmenopausal women were randomised to oestradiol for 12 weeks or placebo, those receiving oestradiol demonstrated greater frontal lobe activity on functional magnetic resonance imaging during verbal and spatial working memory tasks [32]. Finally, in multiple human studies, oestrogen replacement therapy has been demonstrated to improve verbal memory and executive function [27,29].

### 3.2. Evaluating Cognitive Function in Clinical Studies

The gold standard objective measure of cognitive function is neuropsychological testing, however this is labour intensive and can be difficult to implement outside of a research setting. Neuropsychological testing is conducted by trained researchers or neuropsychologists, and involves performing a battery of tests, where more than one test per cognitive domain is employed to heighten the reliability of results. Neuropsychological test results have been shown to correlate with daily functioning: the greater the number of cognitive domains impacted, the greater the impact on daily functioning [33]. In addition, neuropsychological testing can predict functional recovery after a cognitive insult and assess objective responses to treatment [33]. Despite the utility of these tests, changes in performance can occur due to random variation, practice effects or confounders, such as poor sleep, low mood or intercurrent illness, and certain cognitive measures, such as episodic memory and problem solving, are more vulnerable to these effects than other cognitive domains [33].

In contrast, self-reported cognitive function is a clinically significant outcome, which is distinct from objective measures of neuropsychological function. Rather than being associated with objective neuropsychological test results, self-reported cognitive function has been shown to be associated with anxiety, depression and fatigue [34]. There is substantial variability in questionnaires that assess self-reported cognitive function. For example, multiple studies have used only general quality of life measures, while others have used a range of validated questionnaires that are specific to cognitive function. Commonly used measures include the EORTC QLQ-C30, which only contains two questions that pertain to cognitive function, the Functional Assessment of Cancer Therapy Cognitive Function Instrument (FACT-Cog) and the Cognitive Failures Questionnaire [35,36,37]. In addition, a small number of studies have developed their own questionnaires or interviews.

### 3.3. Cognitive Dysfunction Associated with Endocrine Therapy

As shown in Table 1, most articles investigated women with early-stage breast cancer (*n* = 21); one studied women with either early or advanced breast cancer, three investigated those with advanced breast cancer and one investigated women with carcinoma in situ. Three studies examined women who did not have breast cancer but were at high risk of developing breast cancer and were receiving risk reducing endocrine therapy. A wide variety of cognitive domains have been studied using both neuropsychological testing and self-reported questionnaires evaluating cognitive symptoms, as detailed in Table 2. Overall, there is no difference in the global measures of cognition with endocrine therapy [19,38,39,40,41], however impairments are reported in specific domains, including memory [42,43,44], particularly verbal memory [16,42,45,46,47,48,49,50] as opposed to visual memory [16,19,42,45,48,49,50,51] or working memory [16,38,45,47,48,49,51], and fluency [16,47,51]. Five authors found that endocrine therapy was associated with impaired processing speed [43,45,46,47,48], while four reported no difference [16,44,49,50]. Other domains appear not to be affected by the use of endocrine therapy, including attention, executive function, language, motor function, psychomotor efficiency and visuospatial ability.

Two of the largest studies conducted have been multi-centre randomised controlled trials in postmenopausal women. In the Co-STAR trial, Danhauer et al. randomised 1479 women at high risk of developing breast cancer to receive tamoxifen or raloxifene [39]. Annual neuropsychological testing and depression screening tools were completed, and women were followed for up to five years. No difference in the cognitive outcomes was found between the two groups. In the NSABP B-35 trial, 1193 postmenopausal women with hormone receptor positive ductal carcinoma in situ were randomised to tamoxifen or anastrozole [52]. Self-reported questionnaires examining quality of life and depression were completed every six months for six years. Similarly, no difference in self-reported cognitive function was observed between the treatment groups. However, neither of these trials included a control group, so a true difference between treatment and control may have been missed.

**Table 1:** Studies of the impact of endocrine therapy on cognition in women at high risk of breast cancer, with invasive breast cancer or breast carcinoma in situ.

Authors	Population	Study Design	Methods	Results
Bender 2015 [53]	Postmenopausal women with EBC receiving chemotherapy plus anastrozole (*n* = 114), anastrozole (*n* = 173) or control (*n* = 110).	Prospective cohort	Neuropsychological testing performed prior to treatment, and at 6, 12 and 18 months after starting treatment.	Anastrozole was associated with significantly poorer executive function, visual working memory and concentration vs. controls. No differences were noted between groups in verbal memory, mental flexibility, psychomotor efficiency or attention.
Berndt 2016 [42]	Postmenopausal women with EBC receiving tamoxifen alone (*n* = 22), AI only (*n* = 22), switch from tamoxifen to an AI (*n* = 15) and only local therapy (*n* = 21).	Case-control	Neuropsychological testing performed on one occasion.	ET was associated with significantly worse memory scores vs. no systemic therapy. Tamoxifen or tamoxifen followed by AI was associated with significantly improved attention vs. AI alone. No differences in executive function were found between groups.
Biro 2019 [19]	Women with EBC receiving adjuvant tamoxifen (*n* = 13) or AI (*n* = 17) or no ET (*n* = 15).	Prospective cohort	Neuropsychological testing and QoL questionnaire conducted after surgery, then at 6, 12 and 24 months after treatment commencement.	No significant differences in cognition between those receiving ET or observation.
Boele 2015 [16]	Postmenopausal women with EBC receiving adjuvant tamoxifen (*n* = 20), no adjuvant systemic therapy (*n* = 43) or healthy controls (*n* = 44).	Case-control	Neuropsychological testing and questionnaires evaluating cognitive symptoms, depression, anxiety and self-reported HRQoL measured on one occasion.	Tamoxifen was associated with poorer verbal memory and fluency, and worse self-reported cognitive functioning. Higher HRQoL was associated with improved processing and reaction speed.
Buchanan 2015 [14]	2296 women with EBC or in situ breast carcinoma receiving chemotherapy alone (*n* = 288), ET alone (*n* = 822), chemotherapy and ET (*n* = 859) or neither therapy (*n* = 327). Type of ET received was not described.	Case-control	Participants completed a survey via paper or computer assisted telephone interview on one occasion.	ET alone was associated with increased odds of experiencing neurocognitive symptoms (OR 1.64, 95% CI 1.15–2.33) vs. no systemic therapy.
Collins 2009 [45]	Postmenopausal women with EBC receiving tamoxifen (*n* = 31) or anastrozole (*n* = 14) and healthy controls (*n* = 28).	Prospective cohort	Participants underwent neuropsychological testing around the time of commencing ET, and 5–6 months later.	Overall cognition, processing speed and verbal memory declined significantly after commencing either tamoxifen or anastrozole vs. control.
Chen 2014 [46]	Premenopausal women with EBC receiving tamoxifen (*n* = 47), not receiving tamoxifen (*n* = 45) and healthy controls (*n* = 50).	Case-control	Participants performed tasks to assess decision making ability and neuropsychological testing. Participants had received >12 months of tamoxifen prior to testing.	Tamoxifen was associated with significantly impaired memory, information processing performance and impaired decision-making abilities vs. those not receiving tamoxifen, or controls.
Chen 2017 [54]	Premenopausal women with EBC receiving tamoxifen (*n* = 43), only local therapies (*n* = 41) and healthy controls (*n* = 46).	Case-control	Participants completed an attention network test and neuropsychological testing.	Tamoxifen was associated with significant impairments in executive control vs. those not receiving tamoxifen and controls. There were no differences in alertness or orientation between the groups.
Chen 2017 [55]	Premenopausal women with EBC receiving tamoxifen (*n* = 31) and healthy controls (*n* = 32).	Case-control	Participants completed testing of working memory and underwent resting-state fMRI.	Tamoxifen was associated with significant deficits in working memory and lower functional connectivity of the right dorsolateral prefrontal cortex with the right hippocampus vs. healthy controls.
Danhauer 2013 [39]	Postmenopausal women at increased risk of breast cancer randomised to receive tamoxifen (*n* = 727) or raloxifene (*n* = 752).	RCT	Neuropsychological testing, and testing for depression and affect was performed annually.	No difference observed in cognitive test results between tamoxifen and raloxifene.
Ganz 2016 [52]	Postmenopausal women with DCIS or mixed DCIS and LCIS, randomised to receive tamoxifen (*n* = 601) or anastrozole (*n* = 592).	RCT	Symptom checklist assessing cognitive symptoms was completed at baseline, and every 6 months until the end of treatment.	No difference in cognitive symptoms between tamoxifen and anastrozole.
Harbeck 2016 [56]	Pre and postmenopausal women with MBC receiving palbociclib + fulvestrant (*n* = 347) or placebo + fulvestrant (*n* = 174).	RCT	QoL questionnaires were performed on day 1 of cycles 1–4, then day 1 of every other subsequent cycle from cycle 6 and at the end of treatment.	There was no significant difference in cognitive symptoms between the two groups.
Hedayati 2012 [43]	Pre and postmenopausal women with EBC receiving chemotherapy (*n* = 18), ET (*n* = 45; 12 received anastrozole and 33 tamoxifen), no adjuvant medical therapy (*n* = 14), and healthy controls (*n* = 69).	Prospective cohort	Neuropsychological testing conducted at baseline prior to treatment, after surgery, after adjuvant therapy and after an additional 3 months of follow up.	ET was associated with prolonged processing speed, reduction in attention and memory and slower response speed, compared to healthy controls.
Jenkins 2008 [40]	Postmenopausal women at high risk of developing breast cancer receiving anastrozole (*n* = 111) or placebo (*n* = 116).	RCT	Cognitive assessments performed prior to randomisation, at 6 and 24 months. Self-reported cognitive complaints and psychological symptoms were also measured.	No significant differences between the groups for any cognitive tasks.
Kahan 2021 [57]	Postmenopausal women with MBC resistant to AIs receiving palbociclib plus exemestane or fulvestrant (*n*= 268) or capecitabine (*n* = 269).	RCT	QoL questionnaires were measured at baseline, every 2 cycles for the first 7 cycles, then every 3 cycles until end of treatment, and at the 1st post-treatment visit.	Self-reported cognitive functioning was significantly better in palbociclib + ET arm vs. capecitabine (HR 0.70, 95% CI 0.54–0.89).
Kaufman 2020 [58]	Pre and postmenopausal women with MBC who had progressed after ET, randomised to abemaciclib + fulvestrant (*n* = 446) or fulvestrant (*n* = 223).	RCT	QoL questionnaires were measured at baseline, cycle 2, every 2 cycles from 3–13, then every 3 cycles, and 30 days after completing treatment.	Self-reported cognitive functioning was significantly improved in abemaciclib plus fulvestrant arm vs. placebo plus fulvestrant (HR 0.73, 95% CI 0.57–0.92).
Le Rhun 2015 [38]	Postmenopausal women with EBC receiving adjuvant tamoxifen (*n* = 37) or AI (*n* = 37).	RCT	Neuropsychological assessments and self-reported quality of life questionnaires were completed at baseline, before ET and at 6 and 12 months.	No difference between treatments in global cognitive functioning, episodic memory, working memory, psychomotor speed or executive function.
Legault 2009 [41]	Healthy postmenopausal women at increased risk of breast cancer receiving tamoxifen (*n* = 733) or raloxifene (*n* = 765).	RCT	Neuropsychological testing performed at baseline (either before or after starting treatment), and at 1 and 2 year follow up.	No significant differences in cognitive performance between the two groups.
Lejbak 2010 [51]	Postmenopausal women with EBC receiving adjuvant tamoxifen or anastrozole (*n* = 28) and healthy controls (*n* = 37).	Case-control	Neuropsychological testing and self-reported questionnaires completed on 1 occasion, after at least 12 months of ET.	ET was associated with impaired letter fluency, complex visuomotor attention and speeded manual dexterity. No difference noted in verbal or object location memory, spatial ability, confrontation naming, sematic fluency, visual memory and working memory.
Mandelblatt 2020 ^#^ [59]	Postmenopausal women with EBC receiving ET alone (*n* = 249), chemotherapy ± ET (*n* = 99) and healthy controls (*n* = 340). Type of ET was not described.	Prospective cohort	Self-reported cognitive function and questionnaires performed at baseline, 1, 2 and 3 years.	Cognitive problems were significantly more frequent in those receiving chemotherapy or ET vs. controls, but not after adjustment for multiple comparisons.
Mandelblatt 2018 ^#^ [44]	Postmenopausal women with EBC receiving ET alone (*n* = 237; majority AIs), chemotherapy ± ET (*n* = 94) and healthy controls (*n* = 347). Type of ET was not described.	Prospective cohort	Neuropsychological testing and self-reported questionnaires were performed at baseline, and at 1 and 2 years. *ApoE* genotyping was also performed.	ET was associated with significantly less improvement in learning and memory performance at 1 year vs. the other groups, but improved by 2 years. This effect was largely confined to women who were *ApoE ε4+*. No difference in self-reported cognition between ET vs. control.
Merriman 2017 [60]	Postmenopausal women with EBC receiving anastrozole alone (*n* = 158), chemotherapy + anastrozole (*n* = 104), and healthy controls (*n* = 106).	Prospective cohort	Self-reported cognitive function was assessed using questionnaires at baseline prior to any treatment, after completing chemotherapy prior to anastrozole, then 6 monthly for a total of 4 assessments.	No difference in self-reported cognitive symptoms between those receiving ET alone and controls.
Morales 2004 [61]	Postmenopausal women with EBC/MBC randomised to tamoxifen (*n* = 49), AI (*n* = 28), blinded treatment (tamoxifen or letrozole, *n* = 67) or 2nd line AI after tamoxifen (*n* = 20).	RCT	Self-reported questionnaire at baseline, 1 and 3 months.	No difference in self-reported memory difficulties between groups.
Palmer 2008 [47]	Premenopausal women with EBC receiving tamoxifen (*n* = 23) and healthy controls (*n* = 23).	Case-control	Neuropsychological testing performed on 1 occasion after at least 30 days of tamoxifen use.	Tamoxifen was associated with impaired visual memory, verbal fluency, immediate verbal memory, visuo-spatial ability and processing speed compared to controls.
Shilling 2003 [48]	Women with EBC receiving tamoxifen and/or anastrozole (*n* = 94) and healthy controls (*n* = 35).	Case-control	Neuropsychological testing and cognitive questionnaires were performed at one time point.	Verbal memory and processing speed were significantly lower in patients vs. controls. No difference in visual memory or working memory.
Schilder 2010 ^+^ [49]	Postmenopausal women with EBC randomised to tamoxifen (*n* = 80) or exemestane (*n* = 99) and healthy controls (*n* = 120).	RCT	Neuropsychological assessments and questionnaires were performed prior to treatment and after 1 year of treatment.	Tamoxifen was associated with significantly poorer verbal memory and executive function vs. controls. There was no difference in visual memory, information processing speed, manual motor speed, verbal fluency, reaction speed or working memory with tamoxifen vs. controls. Processing speed and executive functioning was significantly lower with tamoxifen vs. exemestane. There were no differences in any cognitive measures between exemestane vs. controls.
Schilder 2012 ^+^ [34]	Postmenopausal women with EBC receiving tamoxifen (*n* = 80) or exemestane (*n* = 99) and healthy controls (*n* = 120).	RCT	Self-reported cognitive functioning assessed at baseline, prior to treatment and after 1 year of treatment.	The prevalence of difficulties in attention and concentration was increased in tamoxifen users at 1 year.
Underwood 2019 [50]	Postmenopausal women with EBC receiving tamoxifen (*n* = 5) or AI (*n* = 37).	Prospective cohort	Neuropsychological testing performed within 14 days of starting ET, and after 1 year of treatment.	Verbal memory performance declined significantly after 1 year of treatment. There was no significant difference in other cognitive functions (visual memory, processing speed, executive function or perceptual reasoning).
Wagner 2020 [62]	Pre and postmenopausal women with EBC and 21-gene recurrence score of 11–25, receiving chemotherapy + ET (*n* = 218) or ET (*n* = 236; 58% AI and 37% tamoxifen initially).	RCT	Questionnaire performed at baseline, 3, 6, 12, 24 and 36 months.	Self-reported cognitive performance was lower in both treatment groups compared to baseline, and more impaired in those receiving chemotherapy + ET vs. ET alone.

Abbreviations: AI, aromatase inhibitor; DCIS, ductal carcinoma in situ; EBC, early stage breast cancer; ET, endocrine therapy; fMRI, functional magnetic resonance imaging; HR, hazard ratio; HRQOL, health-related quality of life; LCIS, lobular carcinoma in situ; MBC, metastatic breast cancer; QoL, quality of life; RCT, and randomised controlled trial. ^#^, these two manuscripts are derived from the same cohort; ^+^, these two manuscripts are derived from the same cohort.

**Table 2:** Impact of endocrine therapy on specific cognitive domains.

Cognitive Domain	Impaired with Endocrine Therapy	No Difference with Endocrine Therapy
Attention and concentration	Bender 2015 [53], Hedayati 2012 [43], Lejbak 2010 [51]	Bender 2015 [53], Chen 2014 [46], Chen 2017 [54], Mandelblatt 2018 [44]
Executive function	Bender 2015 [53], Chen 2014 [46], Chen 2017 [54], Schilder 2010 [49]	Berndt 2016 [42], Boele 2015 [16], Collins 2009 [45], Le Rhun 2015 [38], Mandelblatt 2018 [44], Underwood 2019 [50]
Fluency	Boele 2015 [16], Lejbak 2010 [51], Palmer 2008 [47]	Schilder 2010 [49]
General memory	Berndt 2016 [42], Hedayati 2012 [43], Mandelblatt 2018 [44]	
Language		Collins 2009 [45], Lejbak 2010 [51]
Mental flexibility		Bender 2015 [53]
Motor function	Lejbak 2010 [51]	Boele 2015 [16], Collins 2009 [45], Palmer 2008 [47], Schilder 2010 [49]
Overall cognition	Collins 2009 [45]	Biro 2019 [19], Le Rhun 2015 [38], Danhauer 2013 [39], Jenkins 2008 [40], Legault 2009 [41]
Processing speed	Collins 2009 [45], Chen 2014 [46], Hedayati 2012 [43], Palmer 2008 [47], Shilling 2003 [48]	Boele 2015 [16], Mandelblatt 2018 [44], Schilder 2010 [49], Underwood 2019 [50]
Psychomotor efficiency		Bender 2015 [53], Le Rhun 2015 [38]
Reaction speed	Hedayati 2012 [43]	Boele 2015 [16], Schilder 2010 [49]
Self-reported cognitive functioning	Boele 2015 [16], Buchanan 2015 [14], Schilder 2012 [34], Wagner 2020 [62]	Ganz 2016 [52], Mandelblatt 2020 [59], Mandelblatt 2018 [44], Merriman 2017 [60], Morales 2004 [61]
Verbal memory	Berndt 2016 [42], Boele 2015 [16], Collins 2009 [45], Chen 2014 [46], Palmer 2008 [47], Shilling 2003 [48], Schilder 2010 [49], Underwood 2019 [50]	Bender 2015 [53], Le Rhun 2015 [38], Lejbak 2010 [51]
Visual memory	Palmer 2008 [47]	Berndt 2016 [42], Biro 2019 [19], Boele 2015 [16], Collins 2009 [45], Lejbak 2010 [51], Shilling 2003 [48], Schilder 2010 [49], Underwood 2019 [50]
Visuospatial ability	Palmer 2008 [47]	Biro 2019 [19], Collins 2009 [45], Lejbak 2010 [51]
Working memory	Bender 2015 [53] Chen 2017 [55]	Boele 2015 [16], Collins 2009 [45], Le Rhun 2015 [38], Lejbak 2010 [51], Palmer 2008 [47], Shilling 2003 [19], Schilder 2010 [49]

### 3.4. Frequency of Cognitive Dysfunction

Overall, limited data have described the frequency of objective or subjective cognitive dysfunction associated with endocrine therapy. Objective deficits using neuropsychological testing have been reported to occur in 32% to 64% of persons receiving endocrine therapy [42,45], while self-reported cognitive symptoms have been reported in 45% of patients in one study [14]. The frequency reported varies owing to differences in testing methods, sample population studied, types of endocrine therapies used and confounders, such as prior chemotherapy use or comorbid psychological conditions. Existing data suggest that cognitive symptoms are less frequent with endocrine therapy compared to chemotherapy, for example in a survey of over 2000 women, there was a much stronger association between chemotherapy use and neurocognitive symptoms than endocrine therapy use (adjusted OR 5.63, 95% CI 3.52–9.00 with chemotherapy alone compared to adjusted OR 1.64, 95% CI 1.15–2.33 for endocrine therapy alone) [14]. Few studies have explored how cognitive function differs between different types of endocrine therapy. Using validated neuropsychological tests, Collins et al. (2009) compared 31 women receiving tamoxifen against 14 women receiving anastrozole and 28 healthy controls. They observed that overall cognition and verbal memory was more impaired in those receiving anastrozole compared to tamoxifen, while processing speed was similarly impaired in the two treatment groups compared to controls [45]. Similarly, Berndt et al. (2016) compared those receiving tamoxifen (*n* = 22), an aromatase inhibitor (*n* = 22), local therapy only (*n* = 21) and those switching from tamoxifen to an aromatase inhibitor (*n* = 15), and on post hoc analyses, observed that those receiving aromatase inhibitors performed worst in tests of general memory, verbal memory and attention [42]. In contrast, Schilder et al. (2010) compared those receiving tamoxifen (*n* = 80) against those receiving exemestane (*n* = 99), and noted that tamoxifen use was associated with significantly slower processing speed and executive functioning compared to exemestane, but there was no difference in verbal, visual or working memory, manual motor speed, verbal fluency or reaction speed [49].

Three recent studies assessed the self-reported cognitive function in those receiving CDK4/6 inhibitors combined with endocrine therapy. In the MONARCH 2 trial, which compared abemaciclib plus fulvestrant to placebo plus fulvestrant in women with hormone receptor positive, HER2 negative advanced breast cancer, self-reported that cognitive functioning was significantly improved with abemaciclib plus fulvestrant compared to placebo plus fulvestrant (HR 0.73, 95% CI 0.57–0.92) [58]. Similarly, in the PEARL study, which compared palbociclib plus endocrine therapy to capecitabine in postmenopausal women with hormone receptor positive metastatic breast cancer, self-reported that cognitive functioning was significantly better in the palbociclib plus endocrine therapy arm compared to the capecitabine arm (HR 0.70, 95% CI 0.54–0.89) [57]. In contrast, no difference in cognitive function was observed in the PALOMA 3 trial, which compared palbociclib plus fulvestrant to placebo plus fulvestrant [56]. Each of these trials used two questions from the European Organisation for Research and Treatment of Cancer Quality-of-Life Questionnaire Core-30 (EORTC QLQ-C30) to assess self-reported cognitive symptoms, which only enabled a limited assessment of perceived cognitive function. These studies raise the hypothesis that CDK 4/6 inhibitors may ameliorate the cognitive symptoms of endocrine therapies, but more research using objective neuropsychological testing and longitudinal follow up is required to confirm this.

### 3.5. Risk Factors for Cognitive Dysfunction

Risk factors for cognitive dysfunction with endocrine therapy include lower cognitive reserve, lower educational status, increasing age, ethnicity, depression, fatigue and anxiety [14,34,63,64,65]. In contrast, those with higher education or more demanding jobs are more likely to notice subtle differences in cognitive function and to discuss their cognitive difficulties with health care providers and receive treatment [14]. One article did not find any association between self-reported cognitive dysfunction and menopausal status [62], while another observed an association between treatment induced menopause and self-reported cognitive dysfunction [14].

The *APOE ε4* allele is well known to be associated with an increased risk of developing Alzheimer’s disease [66]. In a longitudinal cohort study of postmenopausal women with early breast cancer receiving either adjuvant chemotherapy and anastrozole or adjuvant anastrozole alone, Koleck et al. (2014) assessed whether there was any correlation with cognitive function and APOE genotype [67]. They observed that in women receiving anastrozole alone, those who were homozygous or heterozygous for the *APOE ε4* allele exhibited significantly poorer executive function, learning and memory after commencing anastrozole. There was no significant interaction between *APOE ε4* and cognition in the chemotherapy plus anastrozole group.

### 3.6. Management of Endocrine Therapy-Associated Cognitive Dysfunction

Given the improvement in mortality attributable to breast cancer, there is increasing research into how to best manage cognitive side effects of cancer therapy in order to improve survivors’ quality of life. Nonpharmacological strategies have the most evidence for treating cognitive dysfunction due to endocrine therapy, however the evidence base is small and extrapolations from research conducted in those with cognitive impairment due to other aetiologies is required. For instance, numerous studies in patients with mild cognitive impairment have demonstrated positive effects of exercise on cognition, with mechanisms involved, including reducing pro-inflammatory cytokines, improving brain derived neurotrophic factor concentrations and improving cerebral blood flow regulation [68]. Similarly, a recent systematic review examined the effect of exercise on cognitive function in patients with mixed early-stage cancers and found that exercise conferred benefit [69]. Benefits were observed with both aerobic and anaerobic exercise, and across a range of intervention types. Most of the studies that reported a benefit with exercise used self-reported measures of cognitive symptoms, which may be biased by expectations of benefit due to the intervention, and only 3 of 10 trials that used neuropsychological testing reported a significant effect of exercise. In addition, few trials examining the impact of exercise assessed cognition as a primary outcome. Data regarding exercise in persons with endocrine therapy-associated cognitive dysfunction are limited. Rogers et al. (2009) performed a randomised controlled trial of a physical activity behaviour change intervention for 41 women with breast cancer who were receiving an aromatase inhibitor or selective oestrogen receptor modulator and observed no difference in self-reported cognitive functions after the intervention [70]. This behaviour change intervention involved regular group sessions with a clinical psychologist, and individual exercise training sessions with an exercise specialist. In contrast, Hartman et al. (2018) conduced a randomised controlled trial of exercise in breast cancer survivors who had previously received chemotherapy and/or radiotherapy. In total, 61 of 87 (70.1%) participants received adjuvant endocrine therapy during the trial. The authors evaluated both self-reported and objective cognitive functioning and observed that the exercise intervention did not affect self-reported cognition, but significantly improved processing speed [71].

Cognitive rehabilitation has shown promise as a potential treatment for cancer-related cognitive impairment. Cognitive rehabilitation refers to behavioural interventions targeted at improving cognitive and functional performance and includes cognitive training and cognitive behavioural therapy [72]. A systematic review of 19 studies of cognitive rehabilitation in any type of cancer (excluding central nervous system malignancies) found that all studies reported improvements on at least one self-reported or objective cognitive measure [72]. Of interest, objective improvements were most often reported in memory, executive function and processing speed (memory and processing speed being vulnerable to endocrine therapy). These benefits have also been confirmed in populations receiving endocrine therapy. For example, Bray et al. (2017) conducted a randomised controlled trial of cognitive rehabilitation in 242 adult cancer survivors with persisting cognitive symptoms. While tumour types other than central nervous system malignancies were included, the majority (89%) of participants had breast cancer. All patients had previously received chemotherapy and 70% of women were receiving endocrine therapy. Participants were randomised to a 15-week home-based intervention or standard care and the primary outcome was differences in the perceived cognitive impairment subscale of the FACT-Cog. The authors observed a significant reduction in perceived cognitive impairment after completing the intervention, which was sustained six months afterwards. Those receiving the intervention also demonstrated significantly lower levels of anxiety, depression, stress and fatigue, and improved quality of life. There was no significant difference in neuropsychological test results at any time point. Similarly, Myers et al. (2020) conducted a non-randomised controlled pilot trial of cognitive rehabilitation in 61 female survivors of breast cancer with cognitive complaints 2 months to 5 years after chemotherapy. In this trial, all subtypes of breast cancer were included, and 38 participants (62.3%) were receiving endocrine therapy during the intervention. They provided a cognitive rehabilitation intervention in a group setting both in-person and via telehealth and assessed self-reported cognitive function. The participants receiving the intervention demonstrated significantly improved self-reported cognitive function at the end of the intervention, which was maintained at 6 and 12 months [73].

There is very limited high-quality data to inform the role of pharmacotherapy in managing cognitive side effects of treatment. A double blind randomised controlled trial evaluated coenzyme Q10, an antioxidant, in 30 women with breast cancer receiving tamoxifen using the self-reported EORTC QLQ-C30 [74]. They observed that coenzyme Q10 significantly improved self-reported cognitive functioning compared to placebo (*p* = 0.023), however the absolute change with treatment between groups was only 1.55 points on a 100-point scale, which is not clinically significant. Other agents, such as donepezil, fluoxetine and cotinine, a metabolite of nicotine, were studied in animal models of cancer-related cognitive impairment associated with chemotherapy and demonstrated benefit [75,76,77]. However, several studies in humans with cancer-related cognitive impairment demonstrated that pharmacotherapy confers no benefit. These include studies of erythropoietin, donepezil and paroxetine [78,79,80,81]. There is mixed evidence regarding psychostimulant use: two studies of modafinil and one of methylphenidate demonstrated improvements in objective cognitive measures [82,83,84], while three studies did not [85,86,87]. Many of these psychostimulant studies assessed fatigue as a primary outcome, rather than cognitive function, and further trials are warranted to confirm whether there is benefit. In addition, it is possible that therapies which are shown to work for chemotherapy-related cognitive dysfunction may not be efficacious for cognitive dysfunction due to endocrine therapy.

Preclinical studies are helping to explore novel means of reducing cognitive dysfunction associated with endocrine therapy. For example, Chen et al. (2013) used mouse models to explore the effects of tamoxifen on neural structure and function [88]. They demonstrated in vitro that exposure to tamoxifen caused cell death of oligodendrocyte and glial precursor cells and oligodendrocytes. They also demonstrated that MEK1/2 inhibition prevented tamoxifen-induced cell death, while simultaneously increasing the sensitivity of a breast cancer cell line to tamoxifen. In vivo, tamoxifen caused cell death in the corpus callosum, and reduced cell division in the corpus callosum, subventricular zone and hippocampal dentate gyrus, and concurrent MEK1/2 inhibition reduced cell death in the corpus callosum, but did not alter cell division in the aforementioned regions. While MEK inhibitors have not been effective in breast cancer to date, conceptually this article highlights that targeted therapies used in combination with endocrine therapy may have unexpected cognitive benefits. Given the close relationship between the PI3K-AKT-mTOR pathway and the RAS-RAF-MEK-ERK pathway, we await data on whether alpelisib, a PI3K inhibitor approved for treatment of metastatic hormone receptor positive breast cancer in combination with fulvestrant, affects cognition.

## 4. Discussion

There remain numerous barriers to optimal diagnosis and treatment of cognitive dysfunction associated with endocrine therapy. Under-diagnosis and under-treatment of endocrine therapy-related cognitive dysfunction is very common. A variety of strategies may assist in improving diagnosis of endocrine therapy-related side effects. These include educating clinicians about the effects of endocrine therapy on cognitive function, and to be particularly aware of this side effect in women who have multiple risk factors for cognitive dysfunction, such as advanced age, low baseline cognitive reserve, low mood or anxiety. Symptoms should be proactively assessed for on a regular basis by clinicians. Given a major barrier to diagnosis is the lack of discussion about cognitive side effects, survivorship clinics can offer a supportive environment for patients to discuss side effects more readily with clinicians. Prior to commencing endocrine therapy, health care providers should also discuss potential cognitive side effects; this discussion may enable patients to recognise and report these side effects more readily. Screening tools can also be of use in improving early detection and treatment and could easily be completed by patients prior to their follow up appointments. However, to date, an optimal screening tool for early detection, nor data on the optimal intervention or benefits of early intervention do not exist. Tools, such as the FACT-Cog, a patient reported outcome measure that takes 15–20 min to complete, are easy to utilise, but given the poor correlation of self-reported deficits with objective cognitive deficits, other more objective methods of testing cognitive domains affected by endocrine therapy should be considered. However, these objective measures require more time and resources.

When women develop cognitive side effects, the severity and nature of deficits should be characterised. Endocrine therapy can have direct effects on cognition by influencing oestradiol signalling, as described above, but also indirect effects on cognition mediated by the presence of other side effects. For instance, endocrine therapy is commonly associated with fatigue, sleep disturbance, depression and anxiety, which may all negatively impact one’s cognitive function. Furthermore, the presence of cognitive dysfunction may negatively influence these side effects. When evaluating patients with complaints of cognitive dysfunction, clinicians therefore need to be cognisant of assessing how these other side effects interplay with the person’s cognitive function and treat these comorbidities appropriately. Referral for formal neuropsychological assessment is recommended where cognitive dysfunction is causing significant impact on quality of life or daily functioning, failing to respond to initial treatment, or if there is diagnostic uncertainty. All women should be recommended to engage in exercise given its likely benefits and proven other benefits. For some women experiencing mild symptoms, education regarding cancer-related cognitive impairment and self-management can suffice. However, for women experiencing severe side effects, an engagement in formal cognitive rehabilitation should be encouraged if available. Ultimately clinicians may need to discuss the risks and benefits associated with stopping endocrine therapy, and for some patients, taking a break from endocrine therapy or changing to an alternative type of endocrine therapy may be warranted.

Further research evaluating the multi-modal interventions, employing both evidence-based cognitive rehabilitation interventions and exercise, would be of great benefit. In addition, the development of a simple screening tool that is sensitive for endocrine therapy-related cognitive dysfunction would be very helpful in aiding early diagnosis and treatment. Ideally, this tool would assess both self-reported cognitive symptoms, and objective measures of cognitive performance, particularly memory. The International Cognition and Cancer Task Force developed a range of recommendations for future research, including advising the use of objective neuropsychological testing, outlining recommended neuropsychological tests and detailing methodology for determining the frequency of cognitive impairment [89]. It is encouraging to see the incorporation of overall quality of life measurements in many of the recent pivotal trials of endocrine therapy, however the incorporation of specific cognitive assessments into the design of new studies is also required to address this important and under-reported treatment associated toxicity. While neuropsychological testing is the gold standard for diagnosis of cognitive impairment, it remains important for future trials to measure patient reported outcomes and functional status, as improvements in these measures may still improve quality of life and daily functioning. Future research exploring the dynamics of endocrine therapy-associated cognitive dysfunction in a longitudinal fashion is required. For instance, it is currently unknown whether endocrine therapy causes permanent changes to brain structure or function, or whether cognition returns to baseline function after cessation of therapy. For some patients, the permanency of cognitive dysfunction may be a significant factor in influencing their willingness to complete therapy. Similarly, further research exploring whether cognitive dysfunction is cumulative is important, given the extended duration of endocrine therapy that is being used at present.

## 5. Conclusions

Cognitive dysfunction associated with endocrine therapy remains underdiagnosed and undertreated, despite the substantial impact it can have on patients’ quality of life, adherence and oncological outcomes. It is important that clinicians proactively seek and treat this side effect in order to optimise treatment outcomes. While the evidence base regarding the treatment options is currently limited, some evidence suggests that there is a benefit with non-pharmacological therapies, specifically exercise and cognitive rehabilitation. For women suffering from these side effects, a risk-benefit analysis is recommended, where practitioners discuss with patients the advantages and disadvantages of ceasing endocrine therapy, balancing the risk of recurrence against the impact of the side effects. Further research into screening tools and management strategies is needed to better diagnose and treat affected patients.

## References

[1] DeSantis C.E., Bray F., Ferlay J., Lortet-Tieulent J., Anderson B.O., Jemal A. (2015). International Variation in Female Breast Cancer Incidence and Mortality Rates. Cancer Epidemiol. Biomarkers Prev..

[2] The Global Cancer Observatory, International Agent for Research on Cancer Breast. https://gco.iarc.fr/today/data/factsheets/cancers/20-Breast-fact-sheet.pdf.

[3] Waks A.G., Winer E.P. (2019). Breast Cancer Treatment: A Review. JAMA.

[4] Wojtyla C., Bertuccio P., Ciebiera M., La Vecchia C. (2021). Breast Cancer Mortality in the Americas and Australasia over the Period 1980–2017 with Predictions for 2025. Biology.

[5] Glassman D., Hignett S., Rehman S., Linforth R., Salhab M. (2017). Adjuvant Endocrine Therapy for Hormone-positive Breast Cancer, Focusing on Ovarian Suppression and Extended Treatment: An Update. Anticancer Res..

[6] Pan H., Gray R., Braybrooke J., Davies C., Taylor C., McGale P., Peto R., Pritchard K.I., Bergh J., Dowsett M. (2017). 20-Year Risks of Breast-Cancer Recurrence after Stopping Endocrine Therapy at 5 Years. N. Engl. J. Med..

[7] Pagani O., Regan M.M., Walley B.A., Fleming G.F., Colleoni M., Láng I., Gomez H.L., Tondini C., Burstein H.J., Perez E.A. (2014). Adjuvant exemestane with ovarian suppression in premenopausal breast cancer. N. Engl. J. Med..

[8] Francis P.A., Pagani O., Fleming G.F., Walley B.A., Colleoni M., Láng I., Gómez H.L., Tondini C., Ciruelos E., Burstein H.J. (2018). Tailoring Adjuvant Endocrine Therapy for Premenopausal Breast Cancer. N. Engl. J. Med..

[9] André F., Ciruelos E., Rubovszky G., Campone M., Loibl S., Rugo H.S., Iwata H., Conte P., Mayer I.A., Kaufman B. (2019). Alpelisib for PIK3CA-Mutated, Hormone Receptor–Positive Advanced Breast Cancer. N. Engl. J. Med..

[10] Baselga J., Campone M., Piccart M., Burris H.A., Rugo H.S., Sahmoud T., Noguchi S., Gnant M., Pritchard K.I., Lebrun F. (2012). Everolimus in postmenopausal hormone-receptor-positive advanced breast cancer. N. Engl. J. Med..

[11] Im S.-A., Lu Y.-S., Bardia A., Harbeck N., Colleoni M., Franke F., Chow L., Sohn J., Lee K.-S., Campos-Gomez S. (2019). Overall Survival with Ribociclib plus Endocrine Therapy in Breast Cancer. N. Engl. J. Med..

[12] Turner N.C., Slamon D.J., Ro J., Bondarenko I., Im S.-A., Masuda N., Colleoni M., DeMichele A., Loi S., Verma S. (2018). Overall Survival with Palbociclib and Fulvestrant in Advanced Breast Cancer. N. Engl. J. Med..

[13] Hortobagyi G.N., Stemmer S.M., Burris H.A., Yap Y.S., Sonke G.S., Hart L., Campone M., Petrakova K., Winer E.P., Janni W. (2021). LBA17_PR—Overall survival (OS) results from the phase III MONALEESA-2 (ML-2) trial of postmenopausal patients (pts) with hormone receptor positive/human epidermal growth factor receptor 2 negative (HR+/HER2−) advanced breast cancer (ABC) treated with endocrine therapy (ET) ± ribociclib (RIB). Ann. Oncol..

[14] Buchanan N.D., Dasari S., Rodriguez J.L., Lee Smith J., Hodgson M.E., Weinberg C.R., Sandler D.P. (2015). Post-treatment Neurocognition and Psychosocial Care Among Breast Cancer Survivors. Am. J. Prev. Med..

[15] Bluethmann S.M., Murphy C.C., Tiro J.A., Mollica M.A., Vernon S.W., Bartholomew L.K. (2017). Deconstructing Decisions to Initiate, Maintain, or Discontinue Adjuvant Endocrine Therapy in Breast Cancer Survivors: A Mixed-Methods Study. Oncol. Nurs. Forum.

[16] Boele F.W., Schilder C.M., de Roode M.L., Deijen J.B., Schagen S.B. (2015). Cognitive functioning during long-term tamoxifen treatment in postmenopausal women with breast cancer. Menopause.

[17] Buwalda B., Schagen S.B. (2013). Is basic research providing answers if adjuvant anti-estrogen treatment of breast cancer can induce cognitive impairment?. Life Sci..

[18] MacLusky N.J., Naftolin F., Goldman-Rakic P.S. (1986). Estrogen formation and binding in the cerebral cortex of the developing rhesus monkey. Proc. Natl. Acad. Sci. USA.

[19] Biro E., Kahan Z., Kalman J., Rusz O., Pakaski M., Irinyi T., Kelemen G., Dudás R., Drotos G., Hamvai C. (2019). Cognitive Functioning and Psychological Well-being in Breast Cancer Patients on Endocrine Therapy. In Vivo.

[20] Nalvarte I., Varshney M., Inzunza J., Gustafsson J. (2021). Estrogen receptor beta and neural development. Vitam. Horm..

[21] Garcia-Segura L.M. (2008). Aromatase in the brain: Not just for reproduction anymore. J. Neuroendocrinol..

[22] Bian C., Zhao Y., Guo Q., Xiong Y., Cai W., Zhang J. (2014). Aromatase inhibitor letrozole downregulates steroid receptor coactivator-1 in specific brain regions that primarily related to memory, neuroendocrine and integration. J. Steroid. Biochem. Mol. Biol..

[23] Kretz O., Fester L., Wehrenberg U., Zhou L., Brauckmann S., Zhao S., Prange-Kiel J., Naumann T., Jarry H., Frotscher M. (2004). Hippocampal synapses depend on hippocampal estrogen synthesis. J. Neurosci..

[24] Daniel J.M., Roberts S.L., Dohanich G.P. (1999). Effects of ovarian hormones and environment on radial maze and water maze performance of female rats. Physiol. Behav..

[25] Luine V.N., Richards S.T., Wu V.Y., Beck K.D. (1998). Estradiol enhances learning and memory in a spatial memory task and effects levels of monoaminergic neurotransmitters. Horm. Behav..

[26] Bimonte H.A., Denenberg V.H. (1999). Estradiol facilitates performance as working memory load increases. Psychoneuroendocrinology.

[27] Zec R.F., Trivedi M.A. (2002). The effects of estrogen replacement therapy on neuropsychological functioning in postmenopausal women with and without dementia: A critical and theoretical review. Neuropsychol. Rev..

[28] Schacter D.L., Wagner A.D. (1999). Medial temporal lobe activations in fMRI and PET studies of episodic encoding and retrieval. Hippocampus.

[29] Maki P.M., Sundermann E. (2009). Hormone therapy and cognitive function. Hum. Reprod. Update.

[30] Eberling J.L., Wu C., Tong-Turnbeaugh R., Jagust W.J. (2004). Estrogen- and tamoxifen-associated effects on brain structure and function. Neuroimage.

[31] Hurria A., Patel S.K., Mortimer J., Luu T., Somlo G., Katheria V., Ramani R., Hansen K., Feng T., Chuang C. (2014). The effect of aromatase inhibition on the cognitive function of older patients with breast cancer. Clin. Breast Cancer.

[32] Joffe H., Hall J.E., Gruber S., Sarmiento I.A., Cohen L.S., Yurgelun-Todd D., Martin K.A. (2006). Estrogen therapy selectively enhances prefrontal cognitive processes: A randomized, double-blind, placebo-controlled study with functional magnetic resonance imaging in perimenopausal and recently postmenopausal women. Menopause.

[33] Harvey P.D. (2012). Clinical applications of neuropsychological assessment. Dialogues Clin. Neurosci..

[34] Schilder C.M., Seynaeve C., Linn S.C., Boogerd W., Beex L.V., Gundy C.M., Nortier J.W., van de Velde C.J., van Dam F.S., Schagen S.B. (2012). Self-reported cognitive functioning in postmenopausal breast cancer patients before and during endocrine treatment: Findings from the neuropsychological TEAM side-study. Psychooncology.

[35] Wagner L.I., Sweet J., Butt Z., Lai J.S., Cella D. (2009). Measuring patient self-reported cognitive function: Development of the Functional Assessment of Cancer Therapy–Cognitive Function Instrument. J. Support Oncol..

[36] Broadbent D.E., Cooper P.F., FitzGerald P., Parkes K.R. (1982). The Cognitive Failures Questionnaire (CFQ) and its correlates. Br. J. Clin. Psychol..

[37] Aaronson N.K., Ahmedzai S., Bergman B., Bullinger M., Cull A., Duez N.J., Filiberti A., Flechtner H., Fleishman S.B., de Haes J.C. (1993). The European Organization for Research and Treatment of Cancer QLQ-C30: A quality-of-life instrument for use in international clinical trials in oncology. J. Natl. Cancer Inst..

[38] Le Rhun E., Delbeuck X., Lefeuvre-Plesse C., Kramar A., Skrobala E., Pasquier F., Bonneterre J. (2015). A phase III randomized multicenter trial evaluating cognition in post-menopausal breast cancer patients receiving adjuvant hormonotherapy. Breast Cancer Res. Treat..

[39] Danhauer S.C., Legault C., Bandos H., Kidwell K., Costantino J., Vaughan L., Avis N.E., Rapp S., Coker L.H., Naughton M. (2013). Positive and negative affect, depression, and cognitive processes in the Cognition in the Study of Tamoxifen and Raloxifene (Co-STAR) Trial. Neuropsychol. Dev. Cogn. B Aging Neuropsychol. Cogn..

[40] Jenkins V.A., Ambroisine L.M., Atkins L., Cuzick J., Howell A., Fallowfield L.J. (2008). Effects of anastrozole on cognitive performance in postmenopausal women: A randomised, double-blind chemoprevention trial (IBIS II). Lancet Oncol..

[41] Legault C., Maki P.M., Resnick S.M., Coker L., Hogan P., Bevers T.B., Shumaker S.A. (2009). Effects of tamoxifen and raloxifene on memory and other cognitive abilities: Cognition in the study of tamoxifen and raloxifene. J. Clin. Oncol..

[42] Berndt U., Leplow B., Schoenfeld R., Lantzsch T., Grosse R., Thomssen C. (2016). Memory and Spatial Cognition in Breast Cancer Patients Undergoing Adjuvant Endocrine Therapy. Breast Care.

[43] Hedayati E., Alinaghizadeh H., Schedin A., Nyman H., Albertsson M. (2012). Effects of adjuvant treatment on cognitive function in women with early breast cancer. Eur. J. Oncol. Nurs..

[44] Mandelblatt J.S., Small B.J., Luta G., Hurria A., Jim H., McDonald B.C., Graham D., Zhou X., Clapp J., Zhai W. (2018). Cancer-Related Cognitive Outcomes Among Older Breast Cancer Survivors in the Thinking and Living With Cancer Study. J. Clin. Oncol..

[45] Collins B., Mackenzie J., Stewart A., Bielajew C., Verma S. (2009). Cognitive effects of hormonal therapy in early stage breast cancer patients: A prospective study. Psychooncology.

[46] Chen X., Li J., Chen J., Li D., Ye R., Zhang J., Zhu C., Tian Y., Wang K. (2014). Decision-making impairments in breast cancer patients treated with tamoxifen. Horm. Behav..

[47] Palmer J.L., Trotter T., Joy A.A., Carlson L.E. (2008). Cognitive effects of Tamoxifen in pre-menopausal women with breast cancer compared to healthy controls. J. Cancer Surviv..

[48] Shilling V., Jenkins V., Fallowfield L., Howell T. (2003). The effects of hormone therapy on cognition in breast cancer. J. Steroid Biochem. Mol. Biol..

[49] Schilder C.M., Seynaeve C., Beex L.V., Boogerd W., Linn S.C., Gundy C.M., Huizenga H.M., Nortier J.W., van de Velde C.J., van Dam F.S. (2010). Effects of tamoxifen and exemestane on cognitive functioning of postmenopausal patients with breast cancer: Results from the neuropsychological side study of the tamoxifen and exemestane adjuvant multinational trial. J. Clin. Oncol..

[50] Underwood E.A., Jerzak K.J., Lebovic G., Rochon P.A., Elser C., Pritchard K.I., Tierney M.C. (2019). Cognitive effects of adjuvant endocrine therapy in older women treated for early-stage breast cancer: A 1-year longitudinal study. Support Care Cancer.

[51] Lejbak L., Vrbancic M., Crossley M. (2010). Endocrine therapy is associated with low performance on some estrogen-sensitive cognitive tasks in postmenopausal women with breast cancer. J. Clin. Exp. Neuropsychol..

[52] Ganz P.A., Cecchini R.S., Julian T.B., Margolese R.G., Costantino J.P., Vallow L.A., Albain K.S., Whitworth P.W., Cianfrocca M.E., Brufsky A.M. (2016). Patient-reported outcomes with anastrozole versus tamoxifen for postmenopausal patients with ductal carcinoma in situ treated with lumpectomy plus radiotherapy (NSABP B-35): A randomised, double-blind, phase 3 clinical trial. Lancet.

[53] Bender C.M., Merriman J.D., Gentry A.L., Ahrendt G.M., Berga S.L., Brufsky A.M., Casillo F.E., Dailey M.M., Erickson K.I., Kratofil F.M. (2015). Patterns of change in cognitive function with anastrozole therapy. Cancer.

[54] Chen X., Li J., Zhang J., He X., Zhu C., Zhang L., Hu X., Wang K. (2017). Impairment of the executive attention network in premenopausal women with hormone receptor-positive breast cancer treated with tamoxifen. Psychoneuroendocrinology.

[55] Chen X., He X., Tao L., Li J., Wu J., Zhu C., Yu F., Zhang L., Zhang J., Qiu B. (2017). The Working Memory and Dorsolateral Prefrontal-Hippocampal Functional Connectivity Changes in Long-Term Survival Breast Cancer Patients Treated with Tamoxifen. Int. J. Neuropsychopharmacol..

[56] Harbeck N., Iyer S., Turner N., Cristofanilli M., Ro J., André F., Loi S., Verma S., Iwata H., Bhattacharyya H. (2016). Quality of life with palbociclib plus fulvestrant in previously treated hormone receptor-positive, HER2-negative metastatic breast cancer: Patient-reported outcomes from the PALOMA-3 trial. Ann. Oncol..

[57] Kahan Z., Gil-Gil M., Ruiz-Borrego M., Carrasco E., Ciruelos E., Muñoz M., Bermejo B., Margeli M., Antón A., Casas M. (2021). Health-related quality of life with palbociclib plus endocrine therapy versus capecitabine in postmenopausal patients with hormone receptor-positive metastatic breast cancer: Patient-reported outcomes in the PEARL study. Eur. J. Cancer.

[58] Kaufman P.A., Toi M., Neven P., Sohn J., Grischke E.M., Andre V., Stoffregen C., Shekarriz S., Price G.L., Carter G.C. (2020). Health-Related Quality of Life in MONARCH 2: Abemaciclib plus Fulvestrant in Hormone Receptor-Positive, HER2-Negative Advanced Breast Cancer After Endocrine Therapy. Oncologist.

[59] Mandelblatt J.S., Zhai W., Ahn J., Small B.J., Ahles T.A., Carroll J.E., Denduluri N., Dilawari A., Extermann M., Graham D. (2020). Symptom burden among older breast cancer survivors: The Thinking and Living with Cancer (TLC) study. Cancer.

[60] Merriman J.D., Sereika S.M., Brufsky A.M., McAuliffe P.F., McGuire K.P., Myers J.S., Phillips M.L., Ryan C.M., Gentry A.L., Jones L.D. (2017). Trajectories of self-reported cognitive function in postmenopausal women during adjuvant systemic therapy for breast cancer. Psychooncology.

[61] Morales L., Neven P., Timmerman D., Christiaens M.R., Vergote I., Van Limbergen E., Carbonez A., Van Huffel S., Ameye L., Paridaens R. (2004). Acute effects of tamoxifen and third-generation aromatase inhibitors on menopausal symptoms of breast cancer patients. Anticancer Drugs.

[62] Wagner L.I., Gray R.J., Sparano J.A., Whelan T.J., Garcia S.F., Yanez B., Tevaarwerk A.J., Carlos R.C., Albain K.S., Olson J.A. (2020). Patient-Reported Cognitive Impairment Among Women with Early Breast Cancer Randomly Assigned to Endocrine Therapy Alone Versus Chemoendocrine Therapy: Results From TAILORx. J. Clin. Oncol..

[63] Bender C.M., Merriman J.D., Sereika S.M., Gentry A.L., Casillo F.E., Koleck T.A., Rosenzweig M.Q., Brufsky A.M., McAuliffe P., Zhu Y. (2018). Trajectories of Cognitive Function and Associated Phenotypic and Genotypic Factors in Breast Cancer. Oncol. Nurs. Forum.

[64] Bender C.M., Sereika S.M., Brufsky A.M., Ryan C.M., Vogel V.G., Rastogi P., Cohen S.M., Casillo F.E., Berga S.L. (2007). Memory impairments with adjuvant anastrozole versus tamoxifen in women with early-stage breast cancer. Menopause.

[65] Tometich D.B., Small B.J., Carroll J.E., Zhai W., Luta G., Zhou X., Kobayashi L.C., Ahles T., Saykin A.J., Clapp J.D. (2019). Pretreatment Psychoneurological Symptoms and Their Association With Longitudinal Cognitive Function and Quality of Life in Older Breast Cancer Survivors. J. Pain Symptom Manag..

[66] Liu C.C., Liu C.C., Kanekiyo T., Xu H., Bu G. (2013). Apolipoprotein E and Alzheimer disease: Risk, mechanisms and therapy. Nat. Rev. Neurol..

[67] Koleck T.A., Bender C.M., Sereika S.M., Ahrendt G., Jankowitz R.C., McGuire K.P., Ryan C.M., Conley Y.P. (2014). Apolipoprotein E genotype and cognitive function in postmenopausal women with early-stage breast cancer. Oncol. Nurs. Forum.

[68] Li C., Zhou C., Li R. (2016). Can Exercise Ameliorate Aromatase Inhibitor-Induced Cognitive Decline in Breast Cancer Patients?. Mol. Neurobiol..

[69] Campbell K.L., Zadravec K., Bland K.A., Chesley E., Wolf F., Janelsins M.C. (2020). The Effect of Exercise on Cancer-Related Cognitive Impairment and Applications for Physical Therapy: Systematic Review of Randomized Controlled Trials. Phys. Ther..

[70] Rogers L.Q., Hopkins-Price P., Vicari S., Markwell S., Pamenter R., Courneya K.S., Hoelzer K., Naritoku C., Edson B., Jones L. (2009). Physical activity and health outcomes three months after completing a physical activity behavior change intervention: Persistent and delayed effects. Cancer Epidemiol. Biomarkers Prev..

[71] Hartman S.J., Nelson S.H., Myers E., Natarajan L., Sears D.D., Palmer B.W., Weiner L.S., Parker B.A., Patterson R.E. (2018). Randomized controlled trial of increasing physical activity on objectively measured and self-reported cognitive functioning among breast cancer survivors: The memory & motion study. Cancer.

[72] Fernandes H.A., Richard N.M., Edelstein K. (2019). Cognitive rehabilitation for cancer-related cognitive dysfunction: A systematic review. Support Care Cancer.

[73] Myers J.S., Cook-Wiens G., Baynes R., Jo M.Y., Bailey C., Krigel S., Klemp J., Asher A. (2020). Emerging From the Haze: A Multicenter, Controlled Pilot Study of a Multidimensional, Psychoeducation-Based Cognitive Rehabilitation Intervention for Breast Cancer Survivors Delivered With Telehealth Conferencing. Arch. Phys. Med. Rehabil..

[74] Hosseini S.A., Zahrooni N., Ahmadzadeh A., Ahmadiangali K., Assarehzadegan M.A. (2020). The Effect of CoQ(10) Supplementation on Quality of Life in Women with Breast Cancer Undergoing Tamoxifen Therapy: A Double-Blind, Placebo-Controlled, Randomized Clinical Trial. Psychol. Res. Behav. Manag..

[75] Lyons L., Elbeltagy M., Bennett G., Wigmore P. (2012). Fluoxetine Counteracts the Cognitive and Cellular Effects of 5-Fluorouracil in the Rat Hippocampus by a Mechanism of Prevention Rather than Recovery. PLoS ONE.

[76] Lim I., Joung H.Y., Yu A.R., Shim I., Kim J.S. (2016). PET Evidence of the Effect of Donepezil on Cognitive Performance in an Animal Model of Chemobrain. Biomed Res. Int..

[77] Iarkov A., Appunn D., Echeverria V. (2016). Post-treatment with cotinine improved memory and decreased depressive-like behavior after chemotherapy in rats. Cancer Chemother. Pharmacol..

[78] O’Shaughnessy J.A., Vukelja S.J., Holmes F.A., Savin M., Jones M., Royall D., George M., Von Hoff D. (2005). Feasibility of quantifying the effects of epoetin alfa therapy on cognitive function in women with breast cancer undergoing adjuvant or neoadjuvant chemotherapy. Clin. Breast Cancer.

[79] Mar Fan H.G., Park A., Xu W., Yi Q.-L., Braganza S., Chang J., Couture F., Tannock I.F. (2009). The influence of erythropoietin on cognitive function in women following chemotherapy for breast cancer. Psycho-Oncology.

[80] Lawrence J.A., Griffin L., Balcueva E.P., Groteluschen D.L., Samuel T.A., Lesser G.J., Naughton M.J., Case L.D., Shaw E.G., Rapp S.R. (2016). A study of donepezil in female breast cancer survivors with self-reported cognitive dysfunction 1 to 5 years following adjuvant chemotherapy. J. Cancer Surviv..

[81] Jean-Pierre P., Mohile S., Morrow G., Figueroa-Moseley C., Berenberg J., Atkins J., Weiss M. (2009). Neuroprotective effect of SSRI among 781 cancer patients receiving chemotherapy: A URCC CCOP Study. J. Clin. Oncol..

[82] Kohli S., Fisher S.G., Tra Y., Adams M.J., Mapstone M.E., Wesnes K.A., Roscoe J.A., Morrow G.R. (2009). The effect of modafinil on cognitive function in breast cancer survivors. Cancer.

[83] Lundorff L.E., Jønsson B.H., Sjøgren P. (2009). Modafinil for attentional and psychomotor dysfunction in advanced cancer: A double-blind, randomised, cross-over trial. Palliat. Med..

[84] Escalante C.P., Meyers C., Reuben J.M., Wang X., Qiao W., Manzullo E., Alvarez R.H., Morrow P.K., Gonzalez-Angulo A.M., Wang X.S. (2014). A randomized, double-blind, 2-period, placebo-controlled crossover trial of a sustained-release methylphenidate in the treatment of fatigue in cancer patients. Cancer J..

[85] Lower E.E., Fleishman S., Cooper A., Zeldis J., Faleck H., Yu Z., Manning D. (2009). Efficacy of dexmethylphenidate for the treatment of fatigue after cancer chemotherapy: A randomized clinical trial. J. Pain Symptom Manag..

[86] Mar Fan H.G., Clemons M., Xu W., Chemerynsky I., Breunis H., Braganza S., Tannock I.F. (2008). A randomised, placebo-controlled, double-blind trial of the effects of d-methylphenidate on fatigue and cognitive dysfunction in women undergoing adjuvant chemotherapy for breast cancer. Support Care Cancer.

[87] Berenson J.R., Yellin O., Shamasunder H.K., Chen C.S., Charu V., Woliver T.B., Sanani S., Schlutz M., Nassir Y., Swift R.A. (2015). A phase 3 trial of armodafinil for the treatment of cancer-related fatigue for patients with multiple myeloma. Support Care Cancer.

[88] Chen H.Y., Yang Y.M., Han R., Noble M. (2013). MEK1/2 inhibition suppresses tamoxifen toxicity on CNS glial progenitor cells. J. Neurosci..

[89] Wefel J.S., Vardy J., Ahles T., Schagen S.B. (2011). International Cognition and Cancer Task Force recommendations to harmonise studies of cognitive function in patients with cancer. Lancet Oncol..

